# A Monoclonal Antibody TrkB Receptor Agonist as a Potential Therapeutic for Huntington’s Disease

**DOI:** 10.1371/journal.pone.0087923

**Published:** 2014-02-04

**Authors:** Daniel Todd, Ian Gowers, Simon J. Dowler, Michael D. Wall, George McAllister, David F. Fischer, Sipke Dijkstra, Silvina A. Fratantoni, Rhea van de Bospoort, Jessica Veenman-Koepke, Geraldine Flynn, Jamshid Arjomand, Celia Dominguez, Ignacio Munoz-Sanjuan, John Wityak, Jonathan A. Bard

**Affiliations:** 1 CHDI Management/CHDI Foundation, Princeton, New Jersey, United States of America; 2 BioFocus, Saffron Walden, United Kingdom; 3 BioFocus, Leiden, Netherlands; 4 Galapagos, Leiden, Netherlands; 5 CHDI Management/CHDI Foundation, Los Angeles, California, United States of America; University of Louisville, United States of America

## Abstract

Huntington’s disease (HD) is a devastating, genetic neurodegenerative disease caused by a tri-nucleotide expansion in exon 1 of the huntingtin gene. HD is clinically characterized by chorea, emotional and psychiatric disturbances and cognitive deficits with later symptoms including rigidity and dementia. Pathologically, the cortico-striatal pathway is severely dysfunctional as reflected by striatal and cortical atrophy in late-stage disease. Brain-derived neurotrophic factor (BDNF) is a neuroprotective, secreted protein that binds with high affinity to the extracellular domain of the tropomyosin-receptor kinase B (TrkB) receptor promoting neuronal cell survival by activating the receptor and down-stream signaling proteins. Reduced cortical BDNF production and transport to the striatum have been implicated in HD pathogenesis; the ability to enhance TrkB signaling using a BDNF mimetic might be beneficial in disease progression, so we explored this as a therapeutic strategy for HD. Using recombinant and native assay formats, we report here the evaluation of TrkB antibodies and a panel of reported small molecule TrkB agonists, and identify the best candidate, from those tested, for *in vivo* proof of concept studies in transgenic HD models.

## Introduction

Huntington’s disease (HD) is a devastating and fatal, autosomal dominant neurodegenerative disease whose etiology is simple but poorly understood. Early HD is characterized by chorea and psychiatric mood and cognitive disturbance deficits, followed by rigidity and dementia later in disease progression, with fatality occurring within 15–20 years of clinical diagnosis [1]–[6].

HD is caused by a tri-nucleotide expansion (cytosine, adenosine and guanosine, (CAG)) in exon 1 of the huntingtin gene [7]. The CAG codon encodes for the expression of the amino acid glutamine (Gln or Q); expansion of the polyglutamine (polyQ) chain on the N-terminus of the huntingtin (HTT) protein beyond 39 repeats affords a mutant form (mHTT) which leads to the onset of disease with complete penetrance. This expanded polyQ mutant form of HTT misfolds and aggregates, which occurs concomitantly with disease progression [8], [9]. However, although HD neuropathology reveals the presence of huntingtin protein inclusions in the nucleus and the cytosol of neurons as well as neuropil [10], it is unclear whether these aggregates confer a neuroprotective or neurotoxic effect [11], [12]. There is no current HD therapeutic that modifies the degenerative process. Current treatments are symptomatic and include neuroleptics, antipsychotics and antidepressants, with motor symptoms being treated with the only approved HD drug, tetrabenazine, a vesicular monoamine transporter (V-MAT) inhibitor.

Tropomyosin-receptor kinase (Trk) receptors (TrkA, TrkB and TrkC) are a family of kinase signaling receptors which regulate the peripheral and central nervous system through their interaction with the neurotrophins that include β-nerve growth factor (NGF), NT3, NT4 and brain-derived neurotrophic factor (BDNF). NGF is the preferred ligand for TrkA, BDNF and NT4 are preferred for TrkB, and NT3 for TrkC; NT3 can also bind TrkA and TrkB with reduced affinity [13]. All neurotrophins bind with lower affinity to the structurally distinct p75 receptor; p75 is reported to contribute to divergent cellular functions which include neuronal apoptosis [14], [15]. Binding of BDNF to TrkB induces receptor dimerization and leads to multiple tyrosine trans-phosphorylation events between the juxtaposed kinase domains that modulate catalytic activity (Tyr^706/707^) and form adapter protein docking sites (Tyr^516^, Tyr^816^) needed for pro-survival signal transduction pathways through the PI3K, PLCγ and MAPK pathways [16].

**Figure 1:**
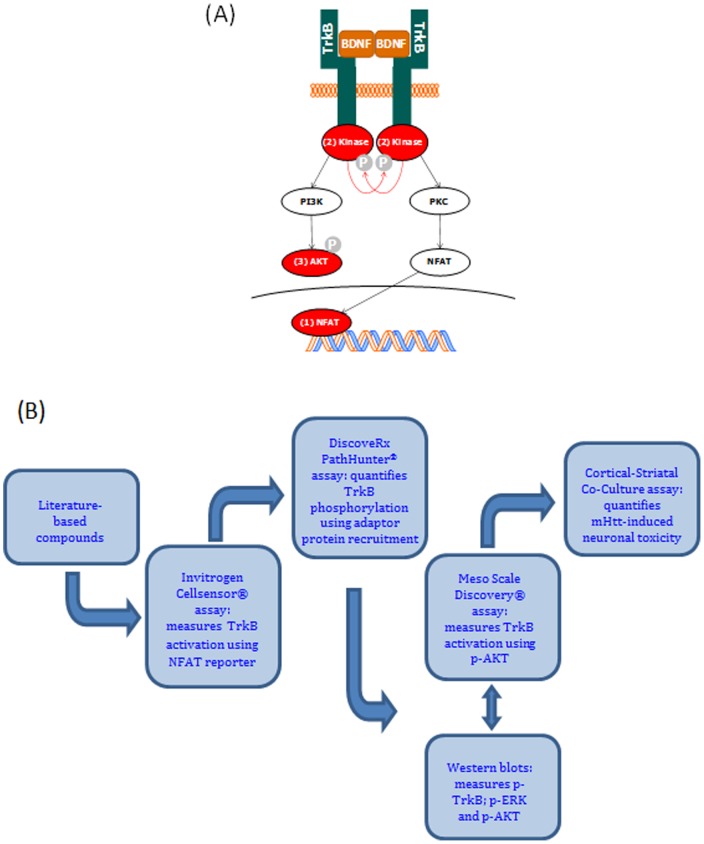
TrkB signaling and assay cascades. (A) TrkB signalling cascade showing proximal and distal assay measurement points. Red arrows represent BDNF-induced trans-phosphorylation events within the intracellular tyrosine kinase domains. Changes in TrkB phosphorylation/activation detected by (1) Invitrogen CellSensor®; (2) DiscoveRx PathHunter®; and (3) MSD® pAKT assays. (B) Screening cascade used to characterize TrkB modulators.

In HD, reduced levels of BDNF and TrkB mRNAs and proteins have been reported in human and mouse model brain cortices; a consequential reduction in neurotrophic support for the striatum has therefore been implicated in disease pathogenesis [17]–[19]. Forebrain knock-out of BDNF in mice results in a striatal expression profile that closely mirrors human HD striatal gene expression [20]. Indeed, over-expression of BDNF in the forebrain reduces the HD phenotype in YAC128 transgenic mice [21]. Poor bioavailability of intrathecally administered BDNF (BDNF precursor protein is 247 amino acids; mature BDNF is 119 amino acids) may underlie the lack of efficacy in clinical studies [22]. We therefore propose that enhancement of TrkB signaling with an exogenously administered TrkB agonist or positive allosteric modulator (PAM) may slow or reverse HD progression. In order to validate and characterize putative TrkB modulators, we applied a number of cell-based assays to measure proximal and distal cell signaling effects (**Figure 1A and 1B**). We also used a TrkB receptor-responsive, rodent primary neuronal HD model of neurodegeneration to confirm that active agents also work on native receptors. As part of our study, we evaluated a comprehensive panel of purported TrkB small molecule agonists that have been published in recent years [**23**] and compared their functionality with two TrkB monoclonal antibody (mAb) agonists [24], [25].

## Materials and Methods

### Ethics Statement

Rat embryos were isolated at The Netherlands Organization for Applied Scientific Research (TNO; Leiden, The Netherlands) before isolation of neurons at Galapagos (Leiden, The Netherlands). Animal experiments were approved by the Institutional Animal Care and Use Committee of TNO (Dierexperimentencommissie TNO), and were in compliance with European Community specifications regarding the use of laboratory animals. All procedures involving animals were conducted humanely and were performed by or under the direction of trained and experienced personnel.

### Culture Media

1x D-MEM with GlutaMax (31966-021), 1x MEM (31095-029), D-MEM/F12 (21331-020), Dialysed Foetal Bovine Serum (dFBS) (26400-044), Horse serum (16050-155), 1M HEPES solution (15630), NEAA (11140), L-glutamine (25030-024), Blasticidin (R210-01), Zeocin (R250-01), Hygromycin B (10687-010), Geneticin (10131-027), Pen/Strep (15140-122), Trypsin/EDTA (25300-054), PBS containing Ca^2+^ and Mg^2+^ (14040) and without Ca^2+^ and Mg^2+^ (14190-094) were obtained from Life Technologies. HyClone II fetal bovine serum (FBS) (SH30066-03) was obtained from PerBio.

### Cell Culture

CellSensor® TrkA-NFAT-bla (K1516), TrkB-NFAT-bla (K1491), TrkC-NFAT-bla (K1515) and NFAT-bla (K1534) CHO-K1 cell lines were obtained from Life Technologies. CellSensor® cells were routinely cultured in growth medium (D-MEM with GlutaMax) supplemented with 10% dFBS, 1x MEM NEAA, 20 mM HEPES, 5 µg/mL Blasticidin and 200 µg/ml Zeocin (No antibiotics for NFAT-bla control line).

PathHunter® U2OS TrkB cell line (93-0463C3) was obtained from DiscoveRx; cells were routinely cultured in growth medium (1x MEM) supplemented with heat inactivated FBS (Hyclone II), 100 µg/mL Hygromycin B and 200 µg/mL Geneticin.

SH-SY5Y human neuroblastoma cells were obtained from ATCC (CRL-2266); cells were routinely cultured in growth medium (D-MEM) supplemented with 10% heat inactivated FBS, 10 mM HEPES, 100 units Pen/strep.

### Reagents

BDNF (PHC7074) and ToxBLAzer reagent (K1136, P36400) were obtained from Life Technologies. NGF-β (N1408), NT-3 (N1905), NT-4 (N1780), and Thapsigargin (T9033) were obtained from Sigma-Aldrich. Tool small molecules: LM22A-4, amitryptiline, 7,8-dihydroxyflavone and N-acetyl serotonin were supplied by CHDI and used after passing QC analysis; B_AG_ peptide was synthesized by Peptide Synthetics; tool antibody panel was sourced commercially (BD mAb #610102; Millipore pAb #07-225) or from Pfizer Inc (38B8 and 29D7 mAbs); a pan-Trk kinase inhibitor was synthesized at BioFocus [26]. All-trans retinoic acid (R2625) and BSA (A0281) were obtained from Sigma-Aldrich. PathHunter® detection kit (93-0001) was obtained from DiscoveRx. DMSO (D/4121/PB17), Triton-X100 (10102913) and Halt Protease and Phosphatase Inhibitor Cocktail (100X) (78444) were obtained from ThermoFisher Scientific. Recombinant pAKT1 (31145) for Meso Scale Discovery studies was obtained from Active Motif; whole cell lysate kit – Phospho (Ser473)/ Total AKT assay (K11100D-2) was obtained from Meso Scale Discovery.

### Western Blotting

Clarified cell lysates were resolved by SDS-PAGE, blotted onto nitrocellulose membranes and immunoprobed with the relevant primary and secondary antibody combinations. Anti-pTrkB Tyr^516^ (4619), anti-pTrkB Tyr^706/707^ (4621), anti-pTrkB, Tyr^816^ (4168), anti-pERK1/2 (4051), anti-pAKT (4051), anti-TrkB (4603), anti-AKT (4685) and anti-ERK1/2 (4695) were sourced from Cell Signalling Technologies. Anti-GAPDH (IMG-5143A) was obtained from Imgenex; Goat anti-rabbit HRP conjugate (PO448) and Goat anti-mouse HRP conjugate (PO447) were sourced from Dako. Goat anti-mouse 800CW conjugate and Goat anti-rabbit 680 conjugate were sourced from LI-COR. Immunoreactive signals were detected using enhanced chemiluminescence reagent (Amersham) or using the Odyssey infra-red reader.

### Invitrogen CellSensor® Trk reporter gene assays

The assays were configured using Invitrogen CHO-K1 cell lines stably expressing a β-lactamase gene under the control of a NFAT promoter region and the relevant Trk (A, B or C) receptor (except for the Trk null cell line). The activity of the β-lactamase enzyme was quantified via FRET with a fluorescent substrate containing a β-lactam ring, according to manufacturer’s provided protocol. All CellSensor® NFAT-bla CHO-K1 cell lines were seeded into black 384w assay plates in CellSensor® plating media (growth media without antibiotics) at 5,000 cells in a volume of 25 µL per well; cells were incubated at 37°C, 5% CO_2_ overnight. Then, the assay plating media was removed and replaced with 32 µL of assay media (plating media without serum) and returned to the incubator for 1 hour. Test compounds were initially titrated in 100% DMSO, then diluted in DMSO/assay media so as to result in a final assay concentration of 0.5% (v/v) DMSO, previously demonstrated not to adversely affect the performance of the assay readouts (data not shown). Test compounds were added (4 µL) and cells were returned to the incubator for 1 hour; dependent on assay mode (agonist, antagonist or positive allosteric modulator) assay media alone (agonist mode), assay media containing EC_80_ neurotrophin (antagonist mode) or assay media containing EC_20_ neurotrophin (PAM mode) were then added to the cells (4 µL) for a further 4 hour incubation. ToxBLAzer detection reagent was reconstituted according to the manufacturer’s instructions and 8 µL/well added to the assay plates, these were then incubated at room temperature protected from light for 2 hours. Levels of β-lactamase activity were then quantified by measuring the fluorescent intensity ratio between ^EX^400nm/^EM^535nm and ^EX^400nm/^EM^460nm and cytotoxicity quantified by measuring the fluorescence intensity at ^EX^590nm/^EM^665nm using a Perkin Elmer EnVision.

### DiscoveRx PathHunter® TrkB Assay

The assay was configured using DiscoveRx PathHunter® U2OS cell line stably co-expressing two cDNAs: a ProLink™ (PK) tagged TrkB receptor and an Enzyme Acceptor (EA) tagged SH2 domain. Activation of the TrkB-PK receptor induces receptor dimerization and trans-phosphorylation, leading to SH2-EA recruitment, and thereby forcing complementation of the two β-galactosidase enzyme fragments (EA and PK). The resulting functional enzyme hydrolyzes substrate to generate a chemiluminescent signal. U2OS-TrkB PathHunter® cells were seeded into white 384w assay plates in plating media (1x MEM; 0.5% v/v horse serum) at 10,000 cells in a volume of 20 µL per well, cells were incubated at 37°C, 5% CO_2_ overnight to adhere to assay plates. Test compounds were added (5 µL) to the cells and the plates were returned to the incubator for 1 hour; dependent on assay mode (agonist, antagonist or positive allosteric modulator) assay media alone (agonist mode), assay media containing EC_80_ BDNF (antagonist mode) or assay media containing EC_20_ BDNF (PAM mode) were then added to the cells (5 µL) for a further 4 hour incubation at room temperature. PathHunter® detection reagent was then added (15 µL/well) and incubated at room temperature protected from light for 1 hour. Levels of β-galactosidase activity were then quantified by measuring the chemiluminescent signal obtained using a Perkin Elmer EnVision.

### Meso Scale Discovery Phospho-AKT Assay

The assay was configured using SH-SY5Y human neuroblastoma cells differentiated with all-trans retinoic acid to induce TrkB expression [27] in combination with the Meso Scale Discovery (MSD) phospho/total AKT detection reagent (K11100D-2). SH-SY5Y cells were seeded at 20,000 cells into Greiner 96-well tissue culture plates in growth media and were incubated at 37°C, 5% CO_2_ for 24 hours. The growth medium was then removed and replaced with growth medium (200 µL) containing 5 µM all-trans retinoic acid and returned to the incubator for an additional 48 hours. The growth medium was then removed and replaced with growth media (200 µL) containing 5 µM all-trans retinoic acid and returned to the incubator for an additional 72 hours. On the day of assay, growth media was removed and replaced with serum-free growth media (90 µL) containing 5 µM all-trans retinoic acid; cells were incubated for 1 hour at 37°C, 5% CO_2_. Test compounds or BDNF contained in serum-free media were added (10 µL) to the cells and the plates were returned to the incubator for 20 minutes; the plate was then placed on ice and 25 µL 5x lysis buffer (100 mM Tris pH 7.4, 250 mM NaCl, 5% v/v Triton X100, 25 mM EDTA, 1x HALT protease/phosphatase inhibitor) was applied to each well. Following a 20 minute incubation on ice the resulting cell lysates (50 µL) were transferred to the MSD AKT assay plate and the manufacturer’s methodology was followed to allow for phospho/total AKT signal detection using the MSD Sector Imager 6000.

### Primary Neuronal mHTT-Induced Cell Death Assay

Cortical and striatal neurons were isolated from E18 rat embryos and separately transfected with a HTT exon1 fragment containing 73 CAG repeats (HTTN90Q73), together with a plasmid encoding for either a green AcGFP or a red AsRed fluorescent reporter before being plated on glial cells and treated with candidate pharmacological agents [28]. After 5 days in culture, the number of fluorescent cells remaining was counted using an automated high content imaging and analysis platform (GE Healthcare InCell 2000) and any changes in neuronal survival quantified.

### Solubility Assay

To align with the functional data, all compound samples and dilutions were prepared using the CellSensor® assay conditions that include: assay buffer, plates, sample volumes and the same timeframe. The assay incubation time was 7 hours. 0, 3 and 7 hour samples were analysed, in triplicate, in the final assay buffer (DMEM/Glutamax + NEAA + 20 mM Hepes pH7.4 + 0.01% BSA, final DMSO concentration 0.5%) at nominal compound concentrations of 5, 20 and 50 µM. 0 hours to assess the solubility at the start of the assay, 3 hours as this is the time-frame over which the compounds exert their activity and 7 hours for the final concentration in the assay. The 7 and 3 hour samples were prepared at times which allowed simultaneous filtering and sample analysis. Samples were analysed by HPLC-UV with confirmation of the peak of interest by LC-MS. All concentrations were determined using a 7 point calibration line prepared in DMSO. Hydrocortisone (good solubility) and reserpine (poor solubility) were included as kinetic solubility assay standards and treated in a similar manner to samples.

## Results

### TrkB Recombinant and Native Cell-Based Screening Cascade

To test and characterize our panel of literature-based TrkB agonists, we established a panel of cell-based assays, shown in **Figure 1B**. We selected the Cellsensor® CHO-K1 TrkB NFAT (nuclear factor of activated T-cells) β-lactamase reporter gene assay [29] as our primary screening assay, which provided a distal transcriptional readout for receptor activation [30]. Trk-null NFAT, TrkA NFAT and TrkC NFAT CHO-K1 cell lines were also used to determine specificity and selectivity properties of the proposed pharmacological tools.

As an orthogonal assay format, we applied the TrkB enzyme fragment complementation (EFC) assay which provided a more proximal readout for receptor activation than the Cellsensor® assay (i.e receptor phosphorylation following activation results in enzyme complementation and activity readout; DiscoveRx PathHunter® assay). We also used additional assay formats to determine TrkB receptor activation status, which included: a) direct measurement of signal transduction phosphorylation events by western blot (phospho/total AKT, phospho/total ERK1/2 and phospho/total TrkB), b) Meso Scale Discovery assay for quantitative measurement of phospho/total AKT in the human neuroblastoma cell line, SH-SY5Y, which expresses endogenous TrkB upon retinoic acid differentiation, and c) an HD relevant primary neuronal mHTT-induced cell death assay, where BDNF is neuroprotective for striatal neurons. All of the assay formats were functionally validated with cognate neurotrophin(s) and a pan-Trk kinase domain inhibitor (data not shown) [26].

### Literature-Based TrkB Functional Antibody Assessment

A panel of four reported TrkB modulating antibodies were tested and showed functional activity in the TrkB NFAT reporter gene assay, agreeing with previously published data (**Table 1**) [24], [25], [31]. The TrkB antibody mediated modulation (agonism and antagonism) observed in this TrkB-dependent NFAT reporter gene assay system further validated the assay format as a viable screening platform for the characterization of putative small molecule TrkB modulators.

**Table 1:** TrkB antibody panel and associated activities.

Antibody	Epitope	Description	Source	Reactivity#	Published hTrkB activity	TrkB NFAT reporter activity
47/TrkB (610102)	Human extracellular subdomains d4 and d5 (TrkB amino acids 156–322)	Mouse monoclonal	Becton Dickinson	Human, Rodent	Partial antagonist; IC_50_ 8.6 nM [31]	IC_50_ 56 nM
pAb (07-225)	Entire rat extracellular domain	Rabbit polyclonal	Millipore	Human, Rodent	Partial agonist; EC_50_ 33 nM [31]	EC_50_ 159 nM
mAb (38B8)	Entire human extracellular domain	Mouse monoclonal	Pfizer Inc	Human, Rodent	Agonist; EC_50_ 5 nM [24]	EC_50_ 34 pM
mAb (29D7)	Entire human extracellular domain	Mouse monoclonal	Pfizer Inc	Human, Rodent	Agonist; EC_50_ 200 pM [25]	EC_50_ 64 pM

# Reactivity as described by suppliers.

### Characterization of Two TrkB Monoclonal Antibody Agonists

**Figure 2:**
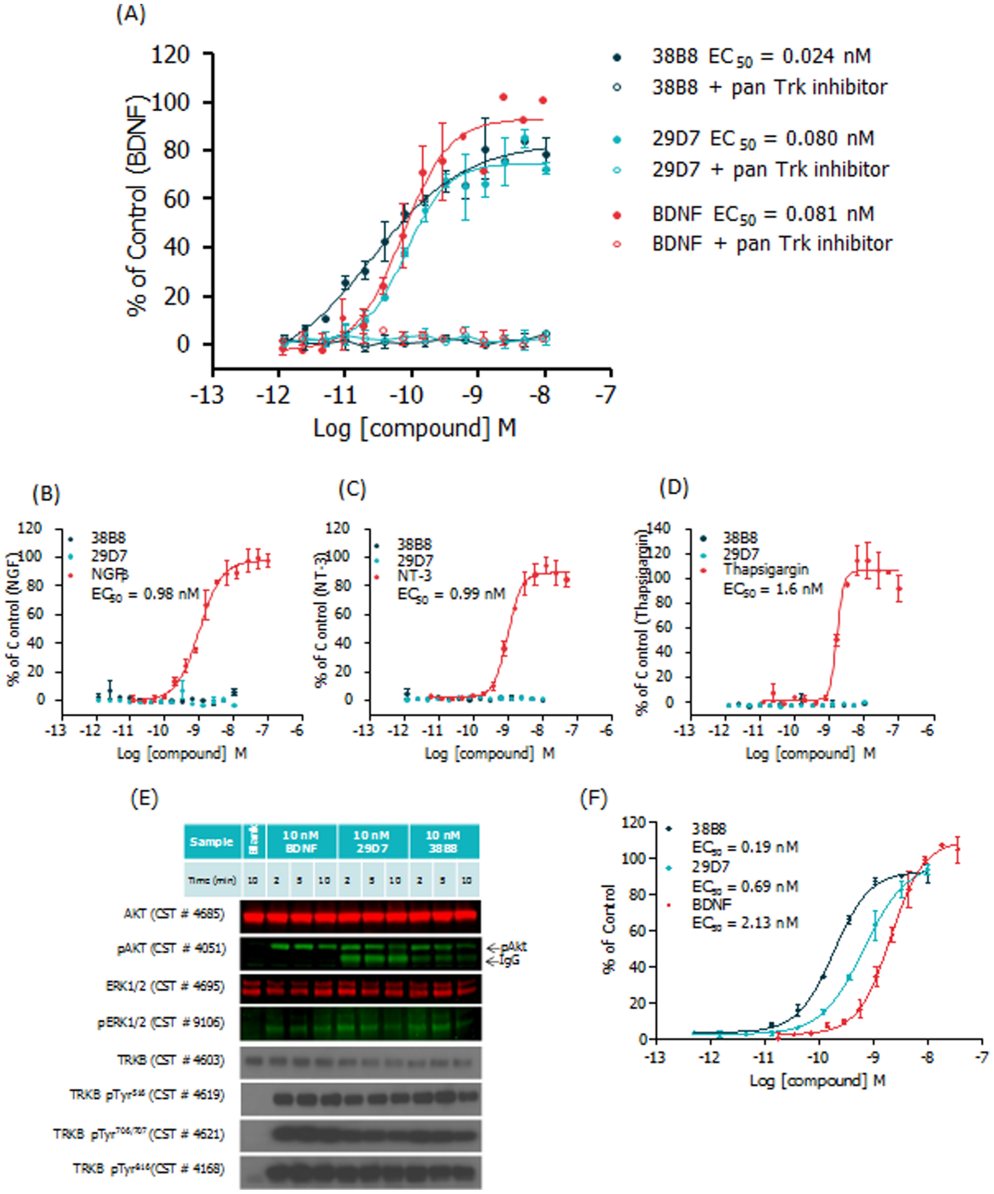
Activation of TrkB signaling pathway by TrkB mAbs. (A) CellSensor® TrkB-NFAT-bla CHO-K1 cells were stimulated with BDNF, mAb 38B8 and mAb 29D7 ± pan-Trk inhibitor (100 nM) over the indicated concentration range for 5 hours before β-lactamase assay was performed as described in Methods. % of control (maximal BDNF concentration  =  9 nM) values were plotted for the indicated concentrations of each ligand (n = 2 ± SEM for each data point). The TrkB mAbs activated TrkB-dependent NFAT signaling that was inhibited by the pan-Trk kinase inhibitor; (B) CellSensor® TrkA-NFAT-bla, (C) CellSensor^®^ TrkC-NFAT-bla, and (D) CellSensor® Trk null-NFAT-bla CHO-K1 cells were stimulated with mAb 38B8, mAb 29D7 and their respective cognate ligands (TrkA:NGFβ; TrkC:NT-3; NFAT:thapsigargin) over the indicated concentration range for 5 hours before β -lactamase assay was performed as described in Methods. % of control (maximal [NGF/NT-3/thapsigargin]) values were plotted for the indicated concentrations of each ligand (n = 2 ± SEM for each data point). Data suggest that the TrkB agonist mAbs are selective and specific; (E) CellSensor® TrkB-NFAT-bla CHO-K1 cells were stimulated with 10 nM BDNF, mAb 38B8 or mAb 29D7 over a short time course (2, 5 and 10 minutes). Cell lysates were resolved by SDS-PAGE, and phosphorylation levels of TrkB, AKT and ERK1/2 were detected using phospho-specific antibodies (pTrkB Tyr516, CST#4619; pTrkB Tyr706/707, CST#4621; pTrkB, Tyr816, CST#4168; pERK1/2, CST#4051; pAKT, CST#4051). Total levels of TrkB, AKT and ERK1/2 were also assessed (TrkB, CST#4603; AKT, CST#4685; ERK1/2, CST#4695). Relevant secondary antibodies were applied and signal was detected with ECL reagent (TrkB) or using the Odyssey infra-red reader (AKT, ERK). Data suggest activation of TrkB phosphorylation signal transduction cascade by TrkB mAbs; and (F) PathHunter® U2OS TrkB cells were stimulated with BDNF, mAb 38B8 and mAb 29D7 over the indicated concentration range for 5 hours before β-galactosidase assay was performed as described in Methods. % of control (maximal BDNF concentration  =  8 nM) values were plotted for the indicated concentrations of each ligand (n = 2 ± SD for each data point). Data indicate activation of TrkB-dependent SH2 recruitment by TrkB mAbs using the TrkB EFC assay.

A concentration-dependent increase in NFAT reporter gene activity, equipotent to BDNF, was observed for the Pfizer mAbs (**Figure 2A**; representative data). Furthermore, induction of NFAT activity by TrkB mAbs was completely prevented by co-incubation with a pan-Trk tyrosine kinase inhibitor [26], confirming that the mAb-mediated increase in NFAT reporter gene activity requires TrkB receptor activation (**Figure 2A**).

Using the TrkA, TrkC and Trk null NFAT reporter gene cell lines, we confirmed that both Pfizer mAbs were selective for TrkB receptors and that NFAT reporter gene activity was not modulated in the absence of the TrkB receptor, demonstrating specificity (**Figure 2B, C, D**). Validity of the TrkA, TrkC and Trk null NFAT reporter gene cell lines was confirmed using the cognate ligands, NGFβ, NT-3 and thapsigargin (an indirect NFAT activator, mediated by increase in cytosolic calcium), respectively.

As both Pfizer mAbs showed potential to become *in vivo* proof of concept tools and, ultimately, HD therapeutic agents, we used our established *in vitro* assay portfolio to further validate them. To provide direct evidence that the mAb-induced NFAT β-lactamase signal was mediated through TrkB, we evaluated proximal TrkB signalling using two methods: a) western blot detection of TrkB phosphorylation status using the TrkB-NFAT-bla CHO-K1 cell line (part of the Invitrogen CellSensor® system) and b) TrkB EFC detection of SH2 recruitment to activated TrkB receptor (part of the DiscoveRx PathHunter® system). TrkB-NFAT-bla CHO-K1 cells treated with Pfizer mAbs or BDNF over a short time course were evaluated by western blot; a panel of total and phospho-specific TrkB, AKT and ERK1/2 antibodies were applied (**Figure 2E**). The immunoblot data clearly show that, as with BDNF, the Pfizer mAbs induced robust phosphorylation of TrkB (Tyr^516^/Tyr^706/707^/Tyr^816^), and the downstream effector kinases AKT (Ser^473^) and ERK1/2 (Thr^202^/Tyr^204^). The ability of the Pfizer mAbs to induce concentration-dependent increases in the proximal TrkB EFC assay signal confirmed their agonistic mode of action (**Figure 2F**). Collectively, these activity profiles suggest that both mAbs are slightly more potent than BDNF, with mAb 38B8 displaying greater potency than mAb 29D7; however, their maximal responses are slightly below that achieved by BDNF (∼70–80% of BDNF response). A similar profile was observed when TrkB-mediated calcium mobilization in TrkB-NFAT-bla CHO-K1 cells (FLIPR-based assay; data not shown) and TrkB-mediated AKT phosphorylation in human neuroblastoma SH-SY5Y cells were measured (MSD-based assay; **Figure S1**). As seen in the TrkB NFAT reporter gene and EFC assay formats, mAb 38B8 was the most potent agonist; in the case of mAb 29D7, our AKT phosphorylation data substantiated functional data published by Qian *et al.* showing 29D7 mediated neurite outgrowth (EC_50_ 470 pM) and survival in SH-SY5Y cells [25].

### Both TrkB mAbs (38B8 and 29D7) Protect Primary Striatal Neurons from mHTT-Induced Cell Death

**Figure 3:**
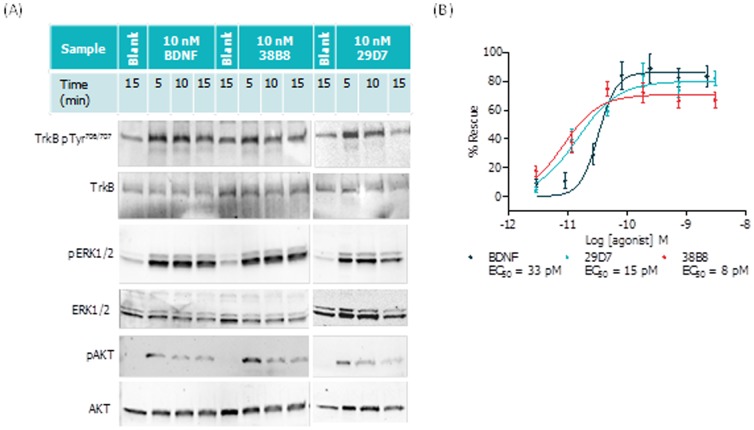
Functional activity of TrkB mAbs in the primary cortico-striatal neuronal co-culture system. Rat primary cortico-striatal co-cultures were stimulated with BDNF, mAb 38B8 and mAb 29D7 and profiled by (A) western blot; agonists tested at 10 nM over the indicated incubation times using non-transfected co-cultures; or (B) mHTT-induced co-culture protocol over the indicated concentration range, as described in the Materials and Methods. For western blot, lysates were prepared and run on SDS-PAGE gels and transferred to membranes that were then hybridized to the corresponding primary antibodies, as described in the Methods and Materials. For mHTT-induced co-culture assay, % Rescue (Normalized to in-plate controls; 0.22 nM BDNF (100% Rescue) and vehicle (0% Rescue)) values were plotted for striatal neurons over the indicated concentrations of each ligand (n = 6 ± SD for each data point). Data demonstrates activation of TrkB phosphorylation signal transduction cascade and rescue of mHTT-induced neuronal toxicity by TrkB mAbs.

BDNF has been shown to protect neurons in a HD mouse model *in vivo* [21], in rat primary neurons expressing HTT *in vitro* [28] and in human HD iPSC-derived neurons *in vitro* [32]. In order to evaluate the TrkB agonist mAbs in an HD context, we assessed their pharmacology using primary neurons in a mutant HTT phenotypic assay. Initially, we confirmed the ability of the TrkB mAbs to activate TrkB and downstream pathways by Western blot in a neuronal system (**Figure 3A**), namely rat primary cortico-striatal co-cultures, as modified from Kaltenbach *et al*. ([28]; also see Materials and Methods).

Next, we used the same co-culture system, but expressing expanded repeat huntingtin (m*HTT*; see Methods) to assess the neuroprotective potential of the TrkB agonist mAbs (**Figure 3B**; representative data). In this co-culture system, mHTT expression, in either the striatal or cortical populations, results in neurotoxicity, as measured by the reduction of the number of surviving, viable cells. As observed with BDNF, both TrkB mAbs reproducibly demonstrated neuroprotection (low picomolar) in striatal neurons (**Figure 3B**), as measured by a significant reduction in cell loss. In contrast, cortical neuronal rescue was less clear for both BDNF and the TrkB mAbs, with protective effects being more variable and showing less potency than observed in striatal neurons (data not shown). We postulated that this may reflect differential TrkB expression between these two neuronal subpopulations. Antibody effects in striatal neurons were confirmed to be on target, as they were reversed in the presence of the pan-Trk kinase inhibitor (data not shown).

### TrkB mAb Mechanism of Action Studies Reveal the Requirement for Antibody Bivalency

**Figure 4:**
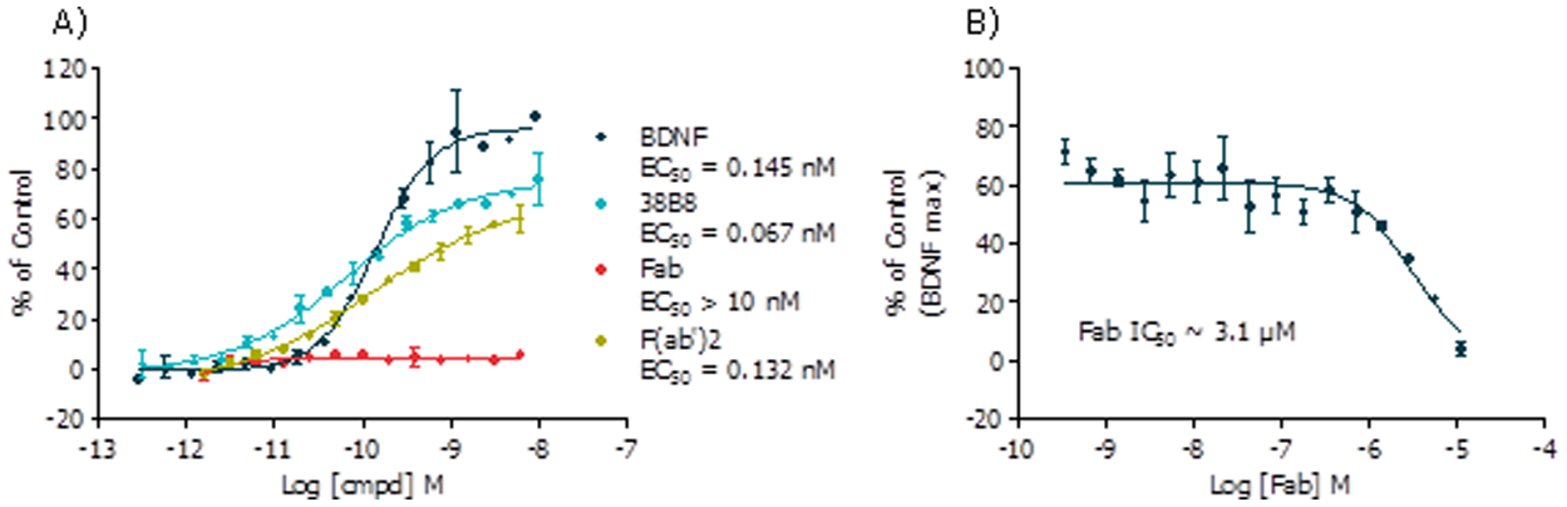
Functional activity of digested 38B8 mAb in the TrkB NFAT reporter gene assays. CellSensor® TrkB-NFAT-bla CHO-K1 cells were stimulated with (A) BDNF, mAb 38B8, 38B8 Fab or 38B8 F(ab’)2 over the indicated concentration range for 5 hours (agonist mode) or (B) 38B8 Fab for 1 hour prior to 0.3 nM BDNF stimulation for 4 hours (antagonist mode) before beta-lactamase assay was performed as described in Methods. % of control (maximal BDNF concentration  =  9 nM) values were plotted for the indicated concentrations of each ligand (n = 2 ± SD for each data point).

Due to the dimeric nature of BDNF and its reported role in mediating TrkB receptor dimerization, we sought to establish whether our functional mAbs required bivalency to exhibit agonism; such a requirement would be consistent with subsequent induction of receptor dimerization being involved in agonism or activation. In order to evaluate this hypothesis, we defragmented the complete IgG1 antibody (38B8) into Fab and F(ab’)_2_ fragments using a commercially available kit (Mouse IgG1 Fab and F(ab´)2 Preparation Kit, following manufacturer’s protocol; Thermo Scientific #44980). If our hypothesis for the mechanism of activation is correct, then the bivalent F(ab’)_2_ fragment derivative would behave as an agonist, whereas the monovalent Fab fragment would be inactive, with the potential to antagonize BDNF-mediated receptor activation should the binding sites overlap. Using the Pierce kit, we prepared the derivative Fab and F(ab’)_2_ fragments from the parent IgG1 antibody (38B8) (**Figure S2**) and assessed their activities in the TrkB NFAT reporter gene assay (**Figure 4**). The bivalent F(ab’)_2_ derivative fragment retained a similar agonist activity (**Figure 4A**) to that of the parent complete IgG1, suggesting that the Fc domain was not required for receptor activation. While the monovalent Fab fragment did not display TrkB agonism, it did, however, show antagonism for BDNF-mediated TrkB activation at low micromolar concentrations (**Figure 4B**), suggesting that bivalency was required for agonism (as reflected by the F(ab’)_2_ fragment) and that the binding site for the antibody overlaped, at least in part, with that of BDNF (as reflected by the Fab fragment).

### Literature-Based TrkB Functional Small Molecule Identification

**Figure 5:**
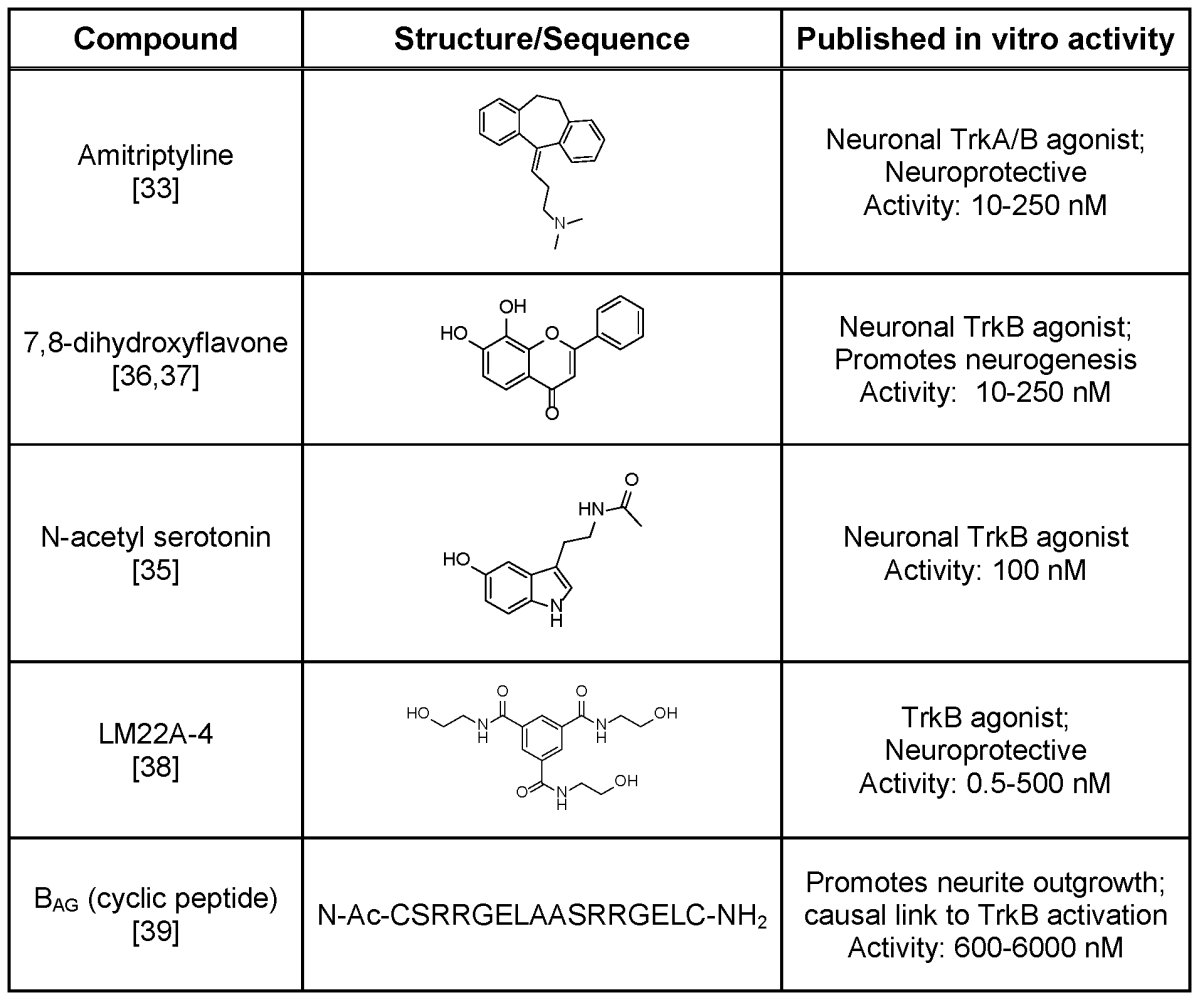
Literature-reported TrkB small molecule agonists. Literature-reporteded TrkB small molecule agonists tested were amitriptyline [33], 7,8-dihydroxyflavone [36], [37], N-acetyl serotonin [35], LM22A-4 [38], and B_AG_ (cyclic peptide [39]. Compound structure or sequence as well as corresponding reported properties and activities are indicated.

We identified literature reported TrkB small molecule agonists (**Figure 5**) for validation and characterization. With the exception of the cyclic peptide (B_AG_; synthesized by Peptide Synthetics), the small molecules were either synthesized using literature routes or procured, where available. The purity of all compounds was assessed by HPLC and ^1^H NMR prior to testing and all showed >95% purity.

Amitriptyline is a tricyclic anti-depressant which has been reported as a potent TrkA and TrkB agonist that promotes receptor heterodimerization [33]. Jang *et al* reported robust TrkB phosphorylation in hippocampal neurons following treatment with 500 nM amitriptyline and state that its activity is ∼100-fold less potent than the cognate neurotrophin. Although known to inhibit the serotonin (SERT) and norepinephrine (NET) transporters, the pharmacology of amitriptyline also encompasses 5-HT1A, 5-HT2A, H1, α-1 and α-2 and muscarinic receptors [34] representing significant off-target activity. *N*-Acetyl serotonin is a natural metabolite accessed through the activity of *N*-acetyl transferase and is a precursor to melatonin, a regulator or circadian rhythms [35]. It is postulated to work in co-operation with BDNF to promote synaptic plasticity and neuroprotection and is reported to activate the TrkB receptor in primary cortical neurons in a dose dependent manner [35]. Jang *et al* report TrkB phosphorylation in hippocampal neurons following treatment with 100 nM N-acetyl serotonin. Similarly, 7,8-dihydroxyflavone, identified in a TrkB-dependent apoptosis assay, was reported as being a TrkB agonist activating AKT and ERK downstream kinases in hippocampal neurons; Liu *et al* report TrkB phosphorylation in primary rat cortical neurons following treatment with 500 nM 7,8-dihydroxyflavone [36], [37]. LM22A-4 was reported as a small molecule BDNF mimetic which prevented neuronal degeneration in rodents; this compound was identified following an *in silico* screen of small molecule libraries with a pharmacophore modelled on the loop 2 domain of BDNF [38]. LM22A-4 (500 nM) was described to selectively activate the TrkB receptor (native and recombinant), as well as the downstream kinases AKT and ERK, in mouse E16 hippocampal neurons and NIH3T3 TrkB-expressing cells. In addition, LM22A-4 was reported to match BDNF efficacy in prevention of neuronal death and improve motor learning after traumatic brain injury. B_AG_ represents a designed 16-mer cyclic peptide based on the SRRGE motif at the amino terminus of NT-4. This neurotrophin mimetic was reported to increase cerebellar neurite outgrowth (maximal effect observed at ∼6 µM) and the activity was attributed to its ability to activate the TrkB receptor [39].

### Putative Small Molecule TrkB Agonist Assessment

**Figure 6:**
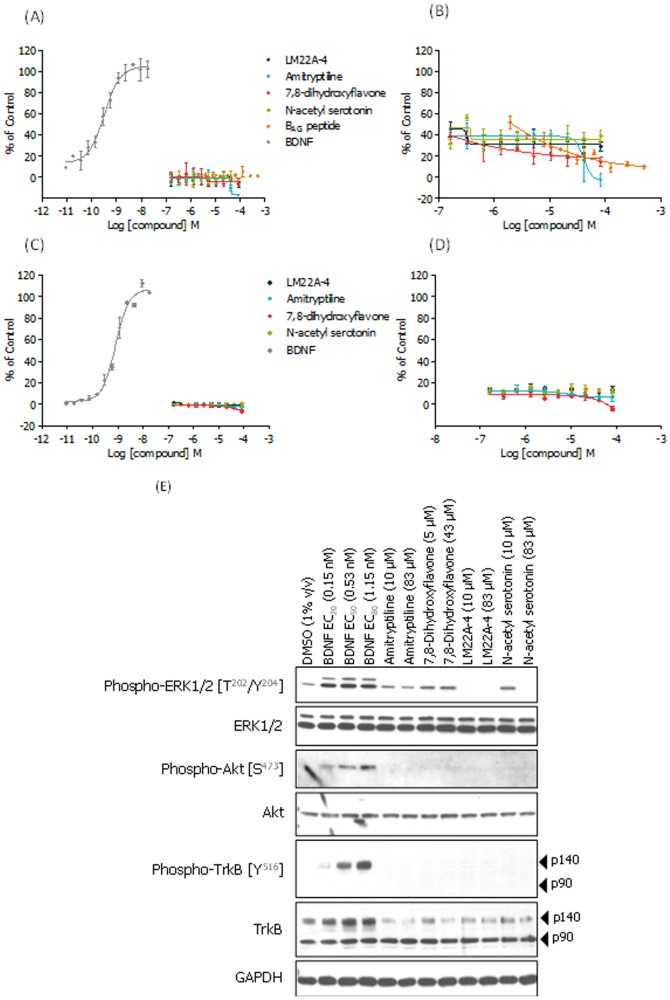
TrkB activation assays: small molecule pharmacology. Literature-claimed TrkB small molecule agonists tested in TrkB agonist (A, C) and PAM (B, D) modes in the CellSensor® NFAT reporter gene (A, B) and PathHunter® EFC (C, D) assays; for PAM mode the compounds are tested in the presence of ∼EC_20_ concentration of BDNF (40 pM, in (B) and 250 pM in (D). For agonist mode, BDNF potency (EC_50_) was 323 pM (A) and 823 pM (C), thereby validating the assay systems. No agonist or PAM activity was observed for the literature-claimed molecules. Curves n = 2 +/– SD, representative data where all experiments were performed in duplicate or triplicate (% of control based on maximal BDNF response). (E) Western blot assessment of ERK1/2, AKT and TrkB phosphorylation status. The CellSensor® TrkB NFAT-bla CHO-K1 cell line was treated for 5 hours using two concentrations of each test compound, as described above. As a positive control for TrkB, ERK1/2 and AKT phosphorylation BDNF was applied at three concentrations (0.15, 0.53 and 1.15 nM). Experiment also configured with a 1 hour treatment regimen; as with 5 hour data, no compound-mediated phosphorylation effects were observed at this earlier time point (data not shown).

Initial activity assessment of the literature-based small molecule panel was carried out using the TrkB NFAT reporter gene assay and compounds were assessed in agonist and PAM modes (**Figure 6A and 6B**, respectively). None of the compounds showed TrkB agonism or PAM activity across a concentration-range that spanned their reported activities. In fact, contrary to its reported activity, amitriptyline showed concentration-dependent TrkB inhibition (**Figure 6B**) indicating an ability to negatively modulate BDNF-induced NFAT reporter gene activation in this assay format.

In order to further investigate the activities (or lack thereof) of the putative literature-claimed TrkB agonist molecules, we also assessed them using the TrkB EFC assay (DiscoveRx PathHunter®), which represents a more proximal readout in the TrkB signalling pathway than the TrkB NFAT reporter gene assay, again employing both agonist and PAM modes (B_AG_ was not characterized further due to having insufficient material). As was observed using the TrkB NFAT reporter gene assay, we did not observe any compound-mediated TrkB agonism or positive allosteric modulation (**Figure 6C, D**).

We were unable to reproduce the reported TrkB activity of any of these literature-based molecules in the TrkB EFC and NFAT reporter gene cell-based assays, reflecting proximal and distal TrkB signalling activities, respectively. In order to assess these molecules further, we used direct measurement of TrkB phosphorylation, and the down-stream kinases ERK1/2 and AKT as an approach to confirm the absence of activity of the TrkB signalling pathway by these literature-based molecules. The TrkB NFAT-bla CHO-K1 cell line was used as a suitable recombinant system. Once again, in agreement with the reporter gene activity, we did not observe molecule-mediated pathway activation (**Figure 6E**) while BDNF treatment induced phosphorylation of TrkB, ERK1/2 and AKT. We speculate that the absence of immunoreactive bands corresponding to basal phospho-ERK1/2 at the low (10 µM) and high (83 µM) concentrations of LM22A-4 and high (83 µM) concentration of *N*-acetyl serotonin (**Figure 6E**) represents compound-mediated and concentration-dependent protein modulation and/or cell toxicity.

Given that the majority of the data previously described for these small molecules was generated in neuronal systems, we performed additional characterization studies of 7,8-dihydroxyflavone and LM22A-4 using our rodent primary neuron based assay system [28], [37], [38].

**Figure 7:**
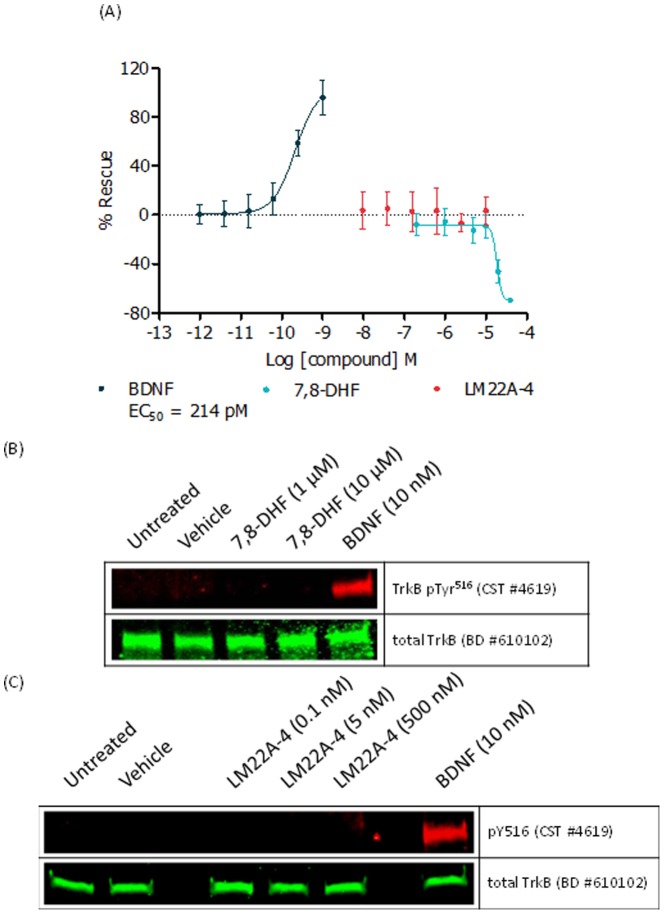
mHTT-induced neuronal toxicity: 7,8-dihydroxyflavone and LM22A-4 pharmacology. Using our primary rat cortico-striatal co-culture system we stimulated with 7,8-dihydroxyflavone, LM22A-4 or BDNF over the indicated concentration range according to the mHTT-induced co-culture protocol (A), as described in Methods. % Rescue (Normalized to in-plate controls; 1 nM BDNF (100% Rescue) and vehicle (0% Rescue)) values were plotted for striatal neurons over the indicated concentrations of each ligand (n = 6 ± SD for each data point). Rat primary cortico-striatal cells (non-transfected) were stimulated with 7,8-dihydroxyflavone (B) or LM22A-4 (C) using the indicated concentrations for 15 minutes and profiled by western blot. BDNF- (10 nM) mediated TrkB phosphorylation validated the experimental system.

We assessed the ability of 7,8-dihydroxyflavone and LM22A-4 to protect rat neurons from mHTT-induced cell death in our cortico-striatal co-culture system and also assessed their ability to modulate TrkB phosphorylation. Both 7,8-dihydroxyflavone and LM22A-4 failed to mimic the ability of BDNF (and the Pfizer mAbs 38B8 and 29D7) to protect striatal neurons from mHTT-induced cell death (**Figure 7A**) or induce TrkB phosphorylation (**Figure 7B, C**); indeed, 7,8-dihydroxyflavone toxicity was evident at concentrations ≥20 µM. These neuronal data in combination with the recombinant TrkB cell line results suggested that these compounds fail to activate the TrkB receptor and don’t display agonist properties in these systems.

## Discussion

A significant body of work indicates that the BDNF-TrkB axis is an attractive target to develop novel therapeutics for HD. One major drawback of BDNF as a therapeutic agent is its poor bioavailability and lipophilic nature [22]. We surmised that intrathecal delivery of BDNF using pumps may not result in broad distribution throughout the striatal target area [40] and elected instead to pursue alternative modalities that would activate TrkB receptors *in vivo*, which included purported small molecule and functional antibody activators of the TrkB receptor.

To enable our characterization of potential TrkB activators, we configured two reporter assay formats which allowed for independent orthogonal readouts of receptor activation. Our primary TrkB assay used a distal readout, an NFAT *β*-lactamase reporter gene assay, which was validated using commercially available BDNF and functional antibodies (**Table 1**), all of which were reported to bind to the TrkB extracellular domain. Our secondary assay format used a more proximal readout, a TrkB functional cell line engineered to co-express a ProLink™ (PK) tagged TrkB receptor and an Enzyme Acceptor (EA) tagged SH2 domain (PathHunter®). Activation of the TrkB-PK tagged receptor induces trans-phosphorylation, leading to SH2-EA recruitment, and forcing complementation of the two *β*-galactosidase enzyme fragments (EA and PK). The resulting functional enzyme hydrolyzes substrate to generate a chemi-luminescent signal. Additional assay formats were also implemented to enable thorough characterization of TrkB modulation, including a primary neuronal system to allow for assessment of native receptors.

We further characterized the Pfizer mAbs using our assay portfolio, and confirmed they are selective and specific for TrkB. The selectivity of these antibodies may have crucial therapeutic advantages over the use of neurotrophins, given that the latter show an ability to also activate the ‘low affinity’ p75^NTR^ receptor that is involved in neuronal cell death, representing an adverse on-target effect [41]. Indeed, we show here that downstream signal transduction for 29D7 and 38B8 mAbs was similar to BDNF, in that they induced TrkB phosphorylation (Y^516^, Y^706,707^, Y^816^), intracellular Ca^2+^ release (data not shown), ERK1/2 and AKT phosphorylation in native and/or recombinant receptor cell-based systems. Furthermore, using these TrkB-specific antibody agonists, we also showed that 29D7 and 38B8 rescued mHTT-induced cell death in a primary neuronal co-culture assay, supporting evaluation of these potentially therapeutic mAbs in a HD proof of concept study. As with BDNF, 29D7 and 38B8 showed preferential rescue of striatal neurons, adding further rationale for their application to HD where striatal cell loss is a major hallmark of the disease.

To characterize its activation mechanism, we proteolytically cleaved 38B8 to generate Fab (monovalent) and F(ab’)_2_ (bivalent) derivative fragments and assessed their functional properties using our TrkB NFAT β-lactamase reporter gene assay. The F(ab’)_2_ derivative fragment displayed agonism with similar potency to that of the parent complete IgG1 (38B8) whereas the monovalent Fab derivative fragment was unable to activate the receptor, highlighting the importance of antibody bivalency for activity and suggesting that the mAb mediates receptor dimerization through its ability to bridge two adjacent receptor molecules. Furthermore, we observed Fab inhibition of BDNF-mediated TrkB activation with potency in the low micromolar range, perhaps indicating that the Fab fragment has much lower binding affinity compared to its bivalent IgG1 and F(ab’)_2_ counterparts. These data also suggest that Fab (and 38B8) bind in a region which overlaps or partially overlaps with the BDNF binding site and such “partial” overlap may also contribute to successful albeit low potency antagonism that was observed.

As with BDNF, the proposed bridging mechanism for 38B8 thought to mediate receptor dimerization and activation is an extremely challenging function for monovalent small molecules. Nevertheless, alternative models of small molecule-mediated dimeric receptor activation have been put forward, including allosteric mechanisms reported for other receptors and support the possibility of similar mechanisms for TrkB [23]. Given the clear therapeutic delivery advantages of optimized small molecules over macromolecular neurotrophins and functional mAbs, we sought to assess a panel of literature-reported TrkB small molecules. We therefore assessed amitriptyline, 7,8-dihydroxyflavone, N-acetyl serotonin, LM22A-4 and a cyclic peptide (B_AG_) that had reported ability to agonise the TrkB receptor in recombinant and/or native cellular systems. Using our recombinant reporter assays, we were unable to detect TrkB agonist or positive allosteric modulatory activities in this panel of small molecules. In contrast to the TrkB agonist activity reported for amitriptyline, we observed inhibitory NFAT reporter gene activity in the micromolar range. To ensure compound availability in the assay, we characterized the solubility profiles in the exact solutions used in our NFAT reporter gene assay (**Table S1**). With the exception of 7,8-dihydroxyflavone, all compounds were soluble at the upper concentration ranges used in our study (5–50 µM). In the case of 7,8-dihydroxyflavone, the solubility profile indicated that only ∼20% of the compound remained in solution beyond the tested lower concentration of 5 µM; regardless, our tests allowed for predicted in-solution concentrations equivalent to and greater than those reported (10–250 nM), thereby suggesting that this compound was genuinely inactive in this TrkB reporter gene system.

To substantiate these initial findings, we assessed the phosphorylation status of the TrkB receptor and its downstream effector kinases following compound treatment in the CHO-K1 TrkB NFAT cell line. In agreement with our reporter assays we failed to observe TrkB, ERK or AKT phosphorylation, in contrast to that observed using BDNF and the 29D7 and 38B8 mAbs.

Given that the majority of reports using these putative TrkB agonists were undertaken in primary neurons, we assessed the activity of two of the most characterized compounds, 7,8-dihydroxyflavone and LM22A-4, in our primary neuronal systems. However, unlike BDNF and the Pfizer mAbs, we did not observe BDNF-TrkB pathway modulation using these small molecules; furthermore, we observed neuronal toxicity for 7,8-dihydroxyflavone at a concentration above 20 µM. Previously, in a TrkB-null cell line (Hippocampal HT-22), 7,8-dihydroxyflavone was reported to protect against glutamate-, hydrogen peroxide- and menadione-induced toxicity while increasing glutathione levels and reducing levels of reactive oxygen species, a profile consistent with an antioxidant effect [42], suggesting its action in any given cellular context may be multi-faceted and likely to involve pathways independent of TrkB. Furthermore, the HT-22 cell viability data presented by Chen *et al.* and our co-culture survival results show similar toxicity effects at concentrations in excess of ∼20 µM. It may have been through alternative pathways that positive effects with 7,8-dihydroxyflavone, were seen in N171-82Z HD mice, as described by Jiang *et al*. [43]. Although the authors demonstrated *in vivo* TrkB phosphorylation at a very modest level, our inability to observe TrkB phosphorylation with this compound is not completely understood but may reflect differences that exist between the complex *in vivo* setting and those described in both engineered tissue culture cells and primary neuronal cultures.

Previous reports indicate that LM22A-4 induced a TrkB signal transduction cascade distinct from that of BDNF [38], in that a modest induction of TrkB-Tyr516 phosphorylation (at ∼30% of the efficacy of BDNF) in hippocampal neurons stimulated a robust up-regulation of ERK and AKT phosphorylation when compared to levels induced by native neurotrophins. This phosphorylation profile was not apparent in our studies using native and recombinant systems. In contrast, we observed no induction of phospho-TrkB (pTyr^516^) in our rat cortico-striatal co-culture system and decreases in basal pERK1/2 in our recombinant NFAT reporter cell line. These latter data suggest that any compound-mediated modulation of pERK levels occur through a mechanism distinct from the BDNF-TrkB pathway and its action is dependent on cellular context and experimental conditions.

Our findings contradict previous reports that purport monovalent small molecules can act as TrkB neurotrophin mimetics or partial agonists and suggest that further research is required to design and identify molecules that will robustly achieve this therapeutic goal. Other possible mechanisms (and screening approaches) for small molecule TrkB receptor modulation could be considered; for example, molecules that increase neurotrophin concentration or receptor expression/trafficking.

Our characterization of reported TrkB small molecule and functional mAb agonists suggests that the 29D7 and 38B8 mAbs from Pfizer are the only candidates tested that, in our hands, induced receptor activation in a manner consistent with the activation profile of the cognate neurotrophin, BDNF. Furthermore, these mAbs showed efficacy in both recombinant and native cellular systems, with the latter system being configured in a HD-relevant context showing mAb-mediated protection of striatal neurons from mHTT-induced cell death.

In the absence of a robust brain penetrant, small molecule agonist, a proof-of-concept evaluation of the 29D7 and 38B8 mAbs in an HD relevant rodent model will be challenging due to the difficulty in delivering the antibody to the striatum. Peripheral mAb injection may also be assessed given a recent publication describing the beneficial effects of systemically administered BDNF in the R6/2 mouse model of HD [44]; however, the anorexigenic side-effect previously reported for peripherally-administered TrkB ligands will need to be carefully considered. Additionally, the mechanism by which peripheral application of BDNF induced striatal and cortex BDNF mRNA levels described was not defined [44] and requires investigation. We are currently considering bilateral microinjection of these mAbs into the striatum and other brain regions in an HD model. The proposed enhancement of TrkB signalling by these mAb TrkB agonists may be beneficial and will provide insight into the tractability of this therapeutic strategy for HD.

## Supporting Information

Figure S1 **Induction of AKT phosphorylation by TrkB mAbs using SH-SY5Y cells expressing endogenous TrkB.** SH-SY5Y cells expressing endogenous TrkB. Retinoic acid-differentiated SH-SY5Y cells were stimulated with BDNF, mAb 38B8 and mAb 29D7 over the indicated concentration range for 20 minutes before measuring % phospho-AKT levels by MSD, as described in Methods. % phosphoprotein  =  ((2* Phospho signal)/(Phospho signal + Total signal)) *100 (n = 2 ± SEM for each data point).
(TIF)

Figure S2 **IgG1 (38B8) digestion profile.** Lane 1 & 8: MW Standards; lanes 2 & 5: IgG1; lanes 3 & 6: F(ab’)_2_; lanes 4 & 7: Fab. 38B8 IgG1 and IgG1 fragments (Fab and F(ab’)_2_) were analyzed by non-reducing (lanes 2–4) and reducing (lanes 5–7) SDS PAGE (4–12% Bis-Tris). Each well was loaded with 1.00–1.25 µg of protein. Coomassie Instant*BLUE* gel stain was used for detection. Expected bands under non-reduced conditions: Fab (45–50 kDa); IgG1 (150 kDa); F(ab’)2 (110 kDa). Expected bands under reduced conditions: Fab, F(ab’)_2_, IgG1 light chain (25 kDa); IgG1 heavy chain (50 kDa). Due to incomplete reduction (lane 5) we also observed a band at ∼100 kDa (most likely representing IgG1 heavy chain dimer).
(TIF)

Table S1 **Solubility assessment.** Solubility analysis of the literature-based small molecules; solubility of the cyclic peptide (BAG) was not determined. Reserpine (poor solubility profile) and hydrocortisone (good solubility profile) were applied as calibration standards.
(DOCX)
