# Supplementary figures and images for: A Monoclonal Antibody TrkB Receptor Agonist as a Potential Therapeutic for Huntington’s Disease
Source: PLoS One. 2014 Feb 4;9(2):e87923. doi: 10.1371/journal.pone.0087923 (PMC3913682; doi:10.1371/journal.pone.0087923)

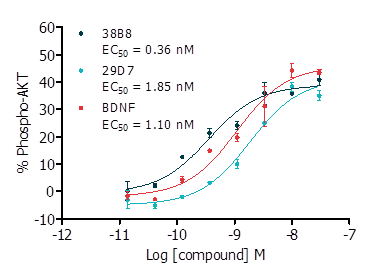

Supplement: Figure S1 — Induction of AKT phosphorylation by TrkB mAbs using SH-SY5Y cells expressing endogenous TrkB. SH-SY5Y cells expressing endogenous TrkB. Retinoic acid-differentiated SH-SY5Y cells were stimulated with BDNF, mAb 38B8 and mAb 29D7 over the indicated concentration range for 20 minutes before measuring % phospho-AKT levels by MSD, as described in Methods. % phosphoprotein = ((2* Phospho signal)/(Phospho signal + Total signal)) *100 (n = 2 ± SEM for each data point). (TIF) [file pone.0087923.s001.tif]

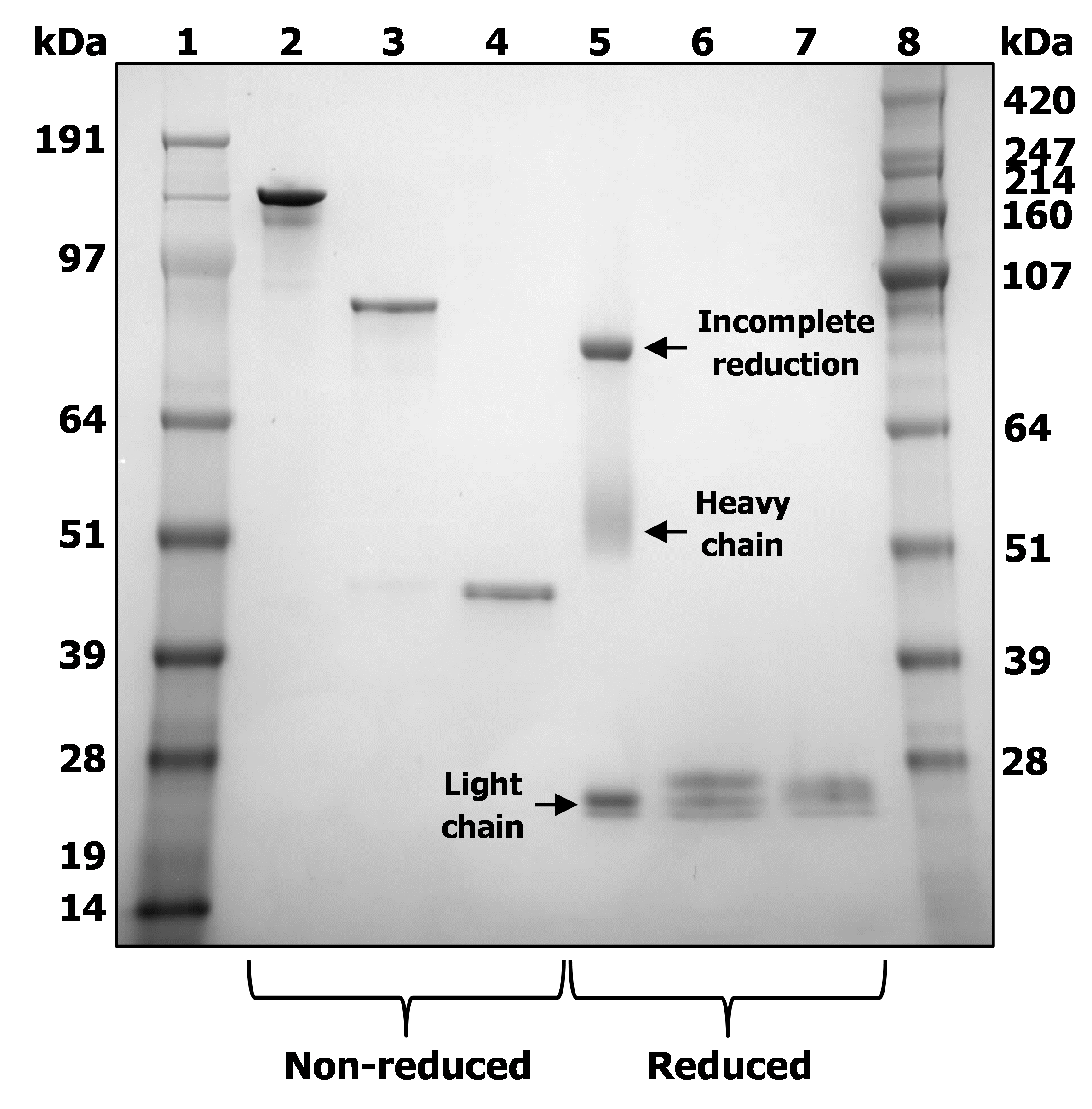

Supplement: Figure S2 — IgG1 (38B8) digestion profile. Lane 1 & 8: MW Standards; lanes 2 & 5: IgG1; lanes 3 & 6: F(ab’)2; lanes 4 & 7: Fab. 38B8 IgG1 and IgG1 fragments (Fab and F(ab’)2) were analyzed by non-reducing (lanes 2–4) and reducing (lanes 5–7) SDS PAGE (4–12% Bis-Tris). Each well was loaded with 1.00–1.25 µg of protein. Coomassie InstantBLUE gel stain was used for detection. Expected bands under non-reduced conditions: Fab (45–50 kDa); IgG1 (150 kDa); F(ab’)2 (110 kDa). Expected bands under reduced conditions: Fab, F(ab’)2, IgG1 light chain (25 kDa); IgG1 heavy chain (50 kDa). Due to incomplete reduction (lane 5) we also observed a band at ∼100 kDa (most likely representing IgG1 heavy chain dimer). (TIF) [file pone.0087923.s002.tif]
