# Supplementary material for: A Monoclonal Antibody TrkB Receptor Agonist as a Potential Therapeutic for Huntington’s Disease
Source: PLoS One. 2014 Feb 4;9(2):e87923. doi: 10.1371/journal.pone.0087923 (PMC3913682; doi:10.1371/journal.pone.0087923)
Supplement: Table S1 — Solubility assessment. Solubility analysis of the literature-based small molecules; solubility of the cyclic peptide (BAG) was not determined. Reserpine (poor solubility profile) and hydrocortisone (good solubility profile) were applied as calibration standards. (DOCX) [file pone.0087923.s003.docx]

**Table S1. Solubility assessment.**

| Compound | Nominal Concentration (µM) | Concentration @ t = 0 (µM) | Concentration @ t = 3 (µM) | Concentration @ t = 7 (µM) |
| --- | --- | --- | --- | --- |
| Amitriptyline | 5 | 3.7 | 4.3 | 3.2 |
|  | 20 | 7.6 | 7.1 | 5.9 |
|  | 50 | 33 | 27 | 27 |
| N-acetyl serotonin | 5 | 7.6 | 8.8 | 10 |
|  | 20 | 13 | 14 | 17 |
|  | 50 | 38 | 44 | 49 |
| 7,8-dihydroxy flavone | 5 | 0.49 | 0.24 | 2.3 |
|  | 20 | 3.0 | 1.9 | 5.2 |
|  | 50 | 13 | 10 | 13 |
| LM22A-4 | 5 | 7.2 | 7.8 | 11 |
|  | 20 | 17 | 17 | 20 |
|  | 50 | 59 | 58 | 64 |
| Hydrocortisone | 5 | 3.4 | 3.4 | 5.5 |
|  | 20 | 11 | 12 | 13 |
|  | 50 | NPD* | 50 | 49 |
| Reserpine | 5 | 2.8 | 3.0 | 3.7 |
|  | 20 | 3.4 | 2.9 | 4.5 |
|  | 50 | 6.7 | 4.5 | 6.9 |

*NPD: No peak detected.

Reserpine (poor solubility profile) and hydrocortisone (good solubility profile) were applied as calibration standards
